# Epoxy–Aminated Lignin Impregnation Combined with Densification for Enhanced Mechanical Properties and Deformation Fixation of Wood

**DOI:** 10.3390/polym17101406

**Published:** 2025-05-20

**Authors:** Zhizun Gao, Jiayi Sun, Zhenke Wei, Fanjun Yu, Zhe Qiu, Zefang Xiao, Yonggui Wang

**Affiliations:** Key Laboratory of Bio-Based Material Science and Technology (Ministry of Education), College of Materials Science and Engineering, Northeast Forestry University, Hexing Road 26, Harbin 150040, China; gaozhizun@nefu.edu.cn (Z.G.);

**Keywords:** aminated lignin, epoxy–amine network, wood densification, mechanical properties

## Abstract

Hot-pressing densification is an effective method to enhance the mechanical properties of wood; however, excessively high pressing temperatures can cause thermal degradation of wood components, compromising these improvements. In this study, aminated lignin (AL), with improved water solubility and reactive amino groups facilitating crosslinking, was utilized as a bio-based amine curing agent for the water-soluble, low-molecular-weight epoxy compound polyethylene glycol diglycidyl ether (PEGDGE). The PEGDGE-AL modifier was applied for wood impregnation, followed by hot-pressing densification at a relatively low temperature of 120 °C, to enhance the mechanical properties of wood. The chemical composition of AL was analyzed using Fourier transform infrared spectroscopy (FTIR), nuclear magnetic resonance spectroscopy (NMR), and X-ray photoelectron spectroscopy (XPS). The gelation and curing behavior of the PEGDGE-AL modifier demonstrated its ability to readily form a network structure at both room temperature and elevated temperatures. The impact strength of densified wood (DW) modified with 12 wt% PEGDGE and 8 wt% AL, denoted as 12PEGDGE+8AL-DW, exhibited an impact strength of 15.2 kJ/m^2^, representing a 72% increase compared to untreated wood (UW). The modulus of rupture (MOR) and modulus of elasticity (MOE) reached 241.1 MPa and 14.6 GPa, respectively, corresponding to 60% and 75% improvements over UW. Furthermore, the 24 h water uptake and thickness swelling of 12PEGDGE+8AL-DW were 45.2% and 24.7%, which were 11% and 43% lower than those of water-impregnated and hot-pressed densified wood (W-DW), respectively. This study provides a low-temperature route for wood densification while contributing to the valorization of lignin in high-performance material applications.

## 1. Introduction

Fast-growing wood is an important renewable resource that has been widely used in the wood industry due to its short growth cycle, wide availability, ease of processing, and sustainability. To reduce pressure on natural forests, many countries are promoting the use of fast-growing plantation wood. For instance, China had over 7.9 million hm^2^ of planted forests by 2018 [1,2]. However, its relatively low density, poor dimensional stability, and insufficient mechanical strength limit its application in high-performance wood products. To overcome these limitations, various modification strategies have been explored [3,4], among which compression densification has proven to be one of the most effective approaches for improving the density and mechanical performance of wood [5,6]. Wood is a natural three-dimensional porous material that can be densified under heat and pressure [7,8]. This densification process reduces the porosity of wood, leading to increased density and improved structural uniformity, which in turn enhances its mechanical properties [9]. However, densification or post heat treatment at elevated temperatures often leads to the thermal degradation of wood components, particularly hemicellulose, resulting in increased brittleness and strength loss [10,11,12]. In addition, high-temperature processing consumes more energy and compromises the overall energy and resource efficiency. To address these challenges, it is necessary to develop densification approaches that operate at lower temperatures, aiming to improve the mechanical performance of fast-growing wood while minimizing thermal degradation of wood and reducing energy consumption.

Chemical impregnation has emerged as a promising route for enhancing the mechanical properties and dimensional stability of wood [4,13,14]. Among various chemical agents, epoxy resins have attracted great attention due to their excellent adhesion, chemical resistance, and ability to form mechanically robust polymer networks [15,16]. In particular, waterborne epoxy resins, which use water as the dispersion medium, offer notable advantages over traditional solvent-based systems by enabling better penetration into wood substrates and reducing environmental impact [17]. Epoxy resins typically consist of reactive epoxy monomers and curing agents [18]. The curing process is significantly accelerated by amine-based curing agents, which initiate crosslinking via ring-opening addition reactions, resulting in the formation of robust polymer networks [16]. The type of amine curing agent critically affects processing behavior [19]. Aliphatic amines are usually highly reactive with epoxy resins and are capable of fast curing, which could pose challenges for wood impregnation modification. In contrast, aromatic amines exhibit lower reactivity due to steric hindrance, thereby avoiding premature curing and enabling deeper resin penetration into the wood structure.

Lignin is the most abundant and renewable aromatic biopolymer in nature and remains highly underutilized [20]. Lignin is mainly derived from black liquor, a by-product generated during the pulping and papermaking process of plant-based fibers. Among the different types of lignin, alkaline lignin—extracted from black liquor—represents the largest fraction [21]. However, its complex and highly polydisperse molecular structure limits its utilization in high-value applications. As a result, most alkaline lignin is currently incinerated for energy recovery, leading to substantial resource underutilization. Notably, lignin molecules contain active functional groups, such as the aliphatic and phenolic hydroxyl groups, which can be chemically modified through various reactions to enhance reactivity and introduce specific functionalities [21,22,23,24]. Among the available strategies, amination has emerged as a promising route to enhance lignin’s chemical reactivity and broaden its range of applications [25,26]. Through the introduction of amino groups, the water solubility and reactivity with epoxy groups of lignin can be greatly improved [27,28]. The resulting aminated lignin (AL) retains the inherent aromatic structure of native lignin, which contributes to improved stiffness and water resistance in cured epoxy networks. Moreover, incorporating the lignin into epoxy systems offers a sustainable pathway for biomass valorization.

**Figure 1:**
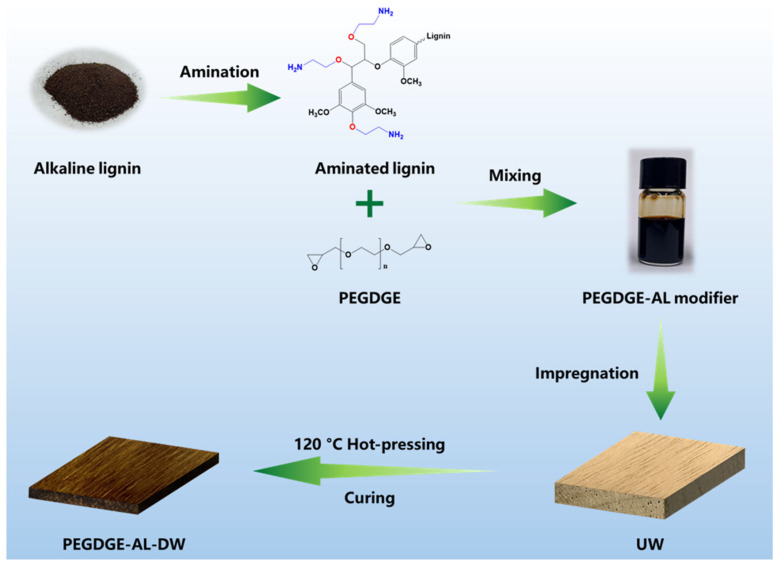
Schematic diagram of the AL synthesis and wood modification process.

In this study, a water-soluble epoxy–aminated lignin compound modifier was applied for wood modification via impregnation combined with hot-pressing densification. The AL was synthesized through aqueous-phase etherification of alkaline lignin with 2-chloroethylamine hydrochloride, a method that avoids the use of toxic organic solvents and enhances the green chemistry profile of the process. The AL was subsequently used as an aromatic amine curing agent for poly (ethylene glycol) diglycidyl ether (PEGDGE), a low-viscosity, water-soluble epoxy monomer, to formulate a reactive impregnation solution. The chemical structure of AL was comprehensively characterized using Fourier transform infrared spectroscopy (FTIR), nuclear magnetic resonance (NMR), and X-ray photoelectron spectroscopy (XPS). The curing behavior of PEGDGE-AL systems at varying AL concentrations was evaluated. Rubberwood specimens were impregnated with the PEGDGE-AL solution and subsequently densified under hot-pressing at a relatively low temperature of 120 °C. This approach aimed to improve the mechanical performance of wood while minimizing thermal degradation and energy consumption. The physical properties, microstructure, dimensional stability, and mechanical performance, including impact strength and flexural strength of the modified wood, were evaluated. The synthesis of AL and wood modification process are illustrated in Figure 1.

## 2. Materials and Methods

### 2.1. Materials and Chemicals

Rubberwood (*Hevea brasiliensis*) specimens with an average oven-dry density of 595 ± 39 kg/m^3^, were sourced from Hainan Province, China. The specimens were cut into dimensions of 50 × 50 × 5 mm^3^ (longitudinal × tangential × radial) for wood modification.

Industrial alkaline lignin was purchased from Feihuang Chemical Co., Ltd. (Xinyi, China). Sodium hydroxide (NaOH) was obtained from Tianli (Tianjin, China). Hydrochloric acid (HCl, 37% aqueous solution) was purchased from Hushi (Shanghai, China). 2-Chloroethylamine hydrochloride (CEH) and Poly (ethylene glycol) diglycidyl ether (PEGDGE) were purchased from Macklin Biochemical Technology Co., Ltd. (Shanghai, China).

### 2.2. Preparation of Aminated Lignin

Industrial alkaline lignin was first purified following the method outlined in a previous study [29]. Aminated lignin (AL) was prepared based on a previously reported method [30] with minor modifications. Briefly, 10 g of purified alkaline lignin was dispersed into 100 mL of 12 wt% NaOH aqueous solution. The mixture was heated to 80 °C with continuous magnetic stirring until the lignin was fully dissolved. Separately, 32.2 g of CEH was dissolved in another 100 mL of 12 wt% NaOH aqueous solution and this solution was subsequently added to the lignin–NaOH mixture. The reaction was kept at 100 °C under reflux with continuous stirring for 12 h. After the reaction, the pH of the mixture was adjusted to 7–8 using 2 mol/L HCl (aqueous solution), leading to the precipitation of the target product. The precipitate was collected by centrifugation and redissolved in 2 mol/L NaOH (aqueous solution). This dissolution–precipitation process was repeated 3–5 times, and the precipitate was dried to obtain purified AL. AL was dissolved in deionized water, with the pH adjusted to 5 using a 2 mol/L HCl aqueous solution and was stored for further use.

### 2.3. Preparation of PEGDGE-AL Modified and Densified Wood

PEGDGE-AL impregnation solutions with varying concentrations of AL were prepared as detailed in Table 1.

The wood specimens were first oven-dried at 103 ± 2 °C and the oven-dried mass and dimensions were measured. The specimens were then impregnated with the prepared PEGDGE-AL solutions under a vacuum of −0.09 MPa for 1 h, followed by a pressure of 0.6 MPa for 4 h. After impregnation, excess solution was gently removed from the specimen surfaces. The specimens were then compressed with a pressure of 8 MPa under room temperature for 10 min using a press (XW-221C, Dongguan Xunwei Testing Machines Co., Ltd., Dongguan, China). The upper and lower plates were subsequently heated to 120 °C, and hot-pressing continued at the same pressure for 1 h. After cooling to room temperature, the compressed specimens were obtained and further dried and cured in an oven at 120 °C for 24 h.

### 2.4. Chemical Analysis of Lignin and AL

The chemical structures of lignin and AL were characterized by Fourier transform infrared (FTIR) spectroscopy and liquid-state ^13^C nuclear magnetic resonance (^13^C NMR) spectroscopy. FTIR spectra were obtained using a Nicolet 6700 spectrometer (Thermo Fisher Scientific, Waltham, MA, USA), and ^13^C NMR measurements were conducted on an AVANCE III HD 500 MHz spectrometer (Bruker, Zurich, Switzerland).

Changes in elemental composition resulting from amination were analyzed using an elemental analyzer (UNICUBE, Elementar, Langenselbold, Germany) and X-ray photoelectron spectroscopy (XPS) using an X-ray photoelectron spectrometer (ESCALAB 250Xi, Thermo Fisher Scientific, USA).

### 2.5. Curing Behavior of PEGDGE-AL Solution

PEGDGE-AL solutions were prepared according to the proportions listed in Table 1 and stored in glass bottles. The gelation behavior at room temperature was evaluated through visual observation. To investigate the curing characteristics, the PEGDGE-AL solutions were thermally cured at 120 °C for 12 h, and the resulting crosslinked products were subsequently observed.

### 2.6. Morphology and Microstructure

Transverse sections of the wood samples were prepared using a microtome. The microstructural features were examined using an ultra-depth-of-field 3D microscope (VHX-6000, Keyence, Osaka, Japan).

### 2.7. Physical Properties

The weight percent gain (WPG) was given by Equation (1):

(1) $$\begin{array}{c} WPG\;\left(\%\right)=\frac{m_{1\;}-\;m_{0}}{m_{0}}\;\times \;100\% \end{array}$$

where *m*_0_ is the oven-dried mass of the unmodified wood specimen; and *m*_1_ is the oven-dried mass of the wood specimen after modification.

The compression ratio (CR) and compression spring back (CSB) were given by Equations (2) and (3):

(2) $$\begin{array}{c} CR\;(\%)=\frac{R_{0}\;-\;{\;R}_{1}}{R_{0}}\;\times \;100 \end{array}$$

(3) $$\begin{array}{c} CSB\;(\%)=\frac{R_{0\;}-\;R_{2}}{R_{0}\;-{\;R}_{1}}\;\times \;100 \end{array}$$

where R_0_ is the radial dimension of the unmodified wood specimen; R_1_ is the radial dimension of the specimen after hot-pressing densification; and R_2_ is the radial dimension of the specimen after densification and curing.

The density of the specimens was calculated according to the mass and volume before and after modification. For each treatment, five replicates were tested.

Surface color changes were assessed using a spectrophotometer (TS7600, 3nh, Shenzhen, China). Color measurements were conducted in the CIELab color space, where the surface color of untreated wood served as the reference for calculating color differences. Each specimen was measured at five different positions, with four replicates per treatment.

### 2.8. Mechanical Properties

Impact strength was measured using a pendulum-type impact tester (XJ-50G, Chengde Mechanical Testing Machine Co., Ltd., Chengde, China). Tests were conducted in an unnotched, simply supported beam configuration with a span of 38 mm and a pendulum energy of 1 J. The specimen dimensions were 50 × 5 × 3 mm (longitudinal × tangential × radial directions) for densified wood and 50 × 5 × 5 mm for untreated wood, reflecting the thickness change caused by hot-pressing. The loading was applied in the radial direction, perpendicular to the growth rings.

Three-point bending tests were conducted following Chinese standard GB/T 9341-2008 [31] using a universal testing machine (RGM-2, Reger Instruments Co., Ltd., Shenzhen, China) with a span of 38 mm and a loading speed of 1 mm/min. The specimen dimensions were 50 × 5 × 3 mm (longitudinal × tangential × radial directions) for densified wood and 50 × 5 × 5 mm for untreated wood, due to the thickness change caused by hot-pressing. The loading was applied in the radial direction, perpendicular to the growth rings. Both modulus of rupture (MOR) and modulus of elasticity (MOE) were determined.

Both the impact test and three-point bending test were performed under oven-dry conditions immediately after specimen preparation. For each group, 10 replicates were tested to enable reliable comparison of the mechanical performance between treated and untreated wood. The results were presented with the box chart.

### 2.9. Dimensional Stability

Due to the variation in thickness of the wood specimens caused by hot-pressing, the wood specimens were cut into dimensions of 25 × 25 mm (longitudinal × tangential) for dimensional stability evaluation, in accordance with the Chinese standard GB/T 17657-2013 [32]. Water uptake (WU) and thickness swelling (TS) were assessed by immersing the wood specimens in deionized water at room temperature for 24 h. The mass and thickness of wood specimens were recorded at specific time intervals: 0.5, 1, 2, 3, 4, 6, 8, 10, 12, and 24 h. WU and TS were given by Equations (4) and (5):

(4) $$\begin{array}{c} WU\;(\%)=\frac{m_{n}\;-\;m_{1}}{m_{1}}\;\times \;100 \end{array}$$

(5) $$\begin{array}{c} TS\;(\%)=\frac{R_{n}\;-{\;R}_{2}}{R_{2}}\;\times \;100 \end{array}$$

where *m*_1_ is the oven-dried mass of the modified wood specimen; *m*_n_ is the instant mass of specimens immersed in water at various intervals; R_2_ is the radial dimension of the modified wood specimen; R_n_ is the instant radial dimension of the specimen immersed in water at various intervals. For each treatment, five replicates were measured.

## 3. Results and Discussion

### 3.1. Chemical Analysis of AL

**Figure 2:**
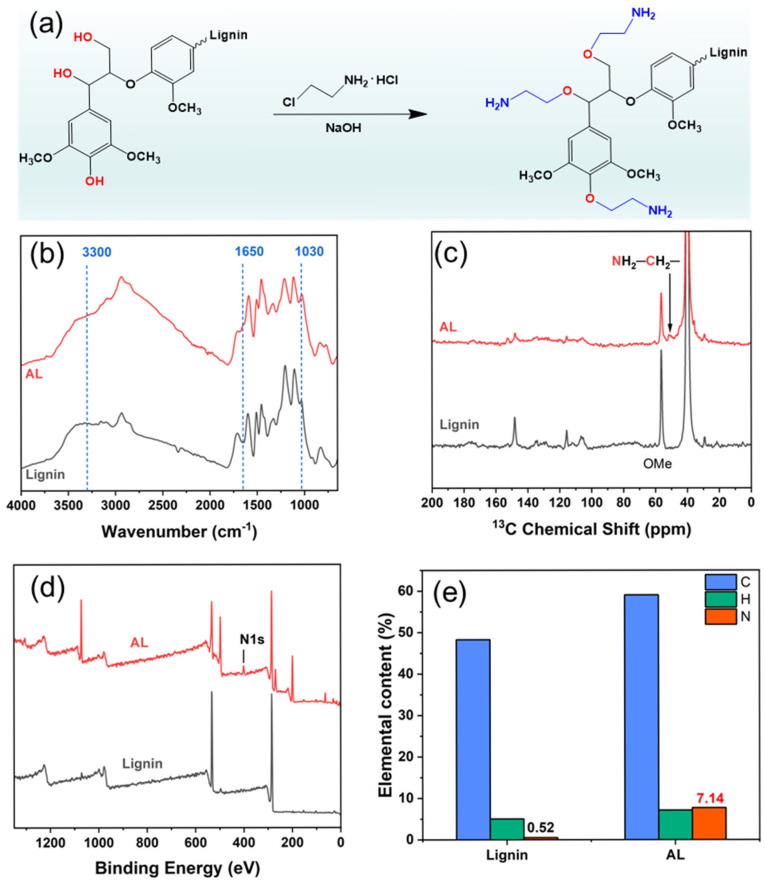
(**a**) Synthesis scheme of AL, (**b**) FTIR spectra of lignin and AL, (**c**) ^13^C NMR of lignin and AL, (**d**) XPS survey spectra of lignin and AL, and (**e**) elemental content of Lignin and AL.

Amination of alkaline lignin was carried out via aqueous-phase etherification using 2-chloroethylamine hydrochloride (CEH) under alkaline conditions. The resulting aminated lignin was purified through 3–5 cycles of dissolution and precipitation, yielding a final product with an approximate yield of 55%. The possible chemical reaction between lignin and CEH is illustrated in Figure 2a. The chemical nature of lignin and AL were analyzed using FTIR. As shown in Figure 2b, the FTIR spectrum of alkaline lignin exhibits characteristic aromatic ring vibration peaks at 1590 and 1505 cm^−1^, corresponding to the stretching vibrations of aromatic C=C bonds [5,33]. After amination, a broad peak located at 3400–3200 cm^−1^ can be assigned to the overlapping stretching vibrations of -OH and -NH_2_ groups [34]. An absorption peak appears at 1650 cm^−1^, corresponding to the N-H stretching vibration of the amino group [26,35], and a peak at 1030 cm^−1^ is associated with the C-O-C stretching vibration of ether linkages [30]. FTIR results provide preliminary evidence for the successful etherification and introduction of amino groups onto lignin. To further confirm the structural changes induced by amination, ^13^C nuclear magnetic resonance (^13^C NMR) spectroscopy was conducted on both lignin and AL. In the ^13^C NMR spectrum of alkaline lignin (Figure 2c), a signal at 56.0 ppm is observed, which is attributed to methoxy (-OCH_3_) carbon atoms [5]. A new peak appears at 50.8 ppm in the spectrum of AL, corresponding to carbon atoms adjacent to amino groups. FTIR and ^13^C NMR results confirm the successful grafting of amino groups onto the lignin structure, thereby verifying the successful preparation of AL.

Figure 2d presents the XPS survey spectra of alkaline lignin and AL. In the spectrum of alkaline lignin, peaks corresponding to carbon (C) and oxygen (O) elements are detected. In contrast, the spectrum of AL exhibits a distinct N1s signal at 400.08 eV, indicating the successful introduction of nitrogen-containing functional groups through amination, which is aligned with the results reported in the literature [33]. Quantitative elemental analysis further corroborates this finding (Figure 2e). The nitrogen content in the alkaline lignin is approximately 0.52%, remaining at a relatively low level. In contrast, in the aminated lignin (AL), it increases markedly to 7.14% following modification. The XPS and quantitative elemental analysis results provide clear evidence of successful amino group (-NH_2_) grafting onto the lignin structure via the etherification reaction.

### 3.2. Gelation Behavior of PEGDGE-AL

**Figure 3:**
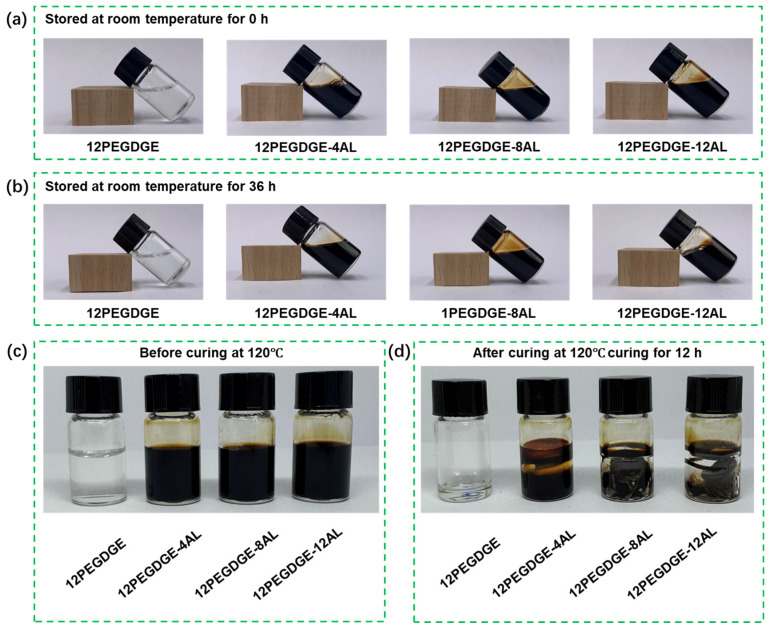
PEGDGE-AL solutions with different AL contents: (**a**) after 0 h and (**b**) 36 h storage at room temperature; (**c**) before and (**d**) after curing at 120 °C for 12 h.

PEGDGE-AL modifiers with varying AL contents were prepared according to Table 1, and the gelation behavior over time was observed (Figure 3). The reference sample containing only 12% PEGDGE remained liquid-state at room temperature and did not undergo gelation. This is attributed to the nature of epoxy groups, which preferentially undergo crosslinking reactions with curing agents such as amines or anhydrides [16]. In the absence of such agents, crosslinking between the epoxy groups of PEGDGE is highly unfavorable under ambient conditions. Upon the addition of AL, the gelation behavior of the PEGDGE-AL modifiers changed significantly. The 12PEGDGE-4AL solution remained an aqueous solution even after storing for 36 h at room temperature. In contrast, 12PEGDGE-8AL and 12PEGDGE-12AL exhibited gradually increasing viscosity after 36 h, indicating the onset of slow crosslinking. This suggests that a higher AL content introduces more reactive amino groups into the system, thereby accelerating gelation. All four solutions demonstrated sufficient stability at room temperature for at least 36 h, ensuring adequate time for wood impregnation. However, the observed gradual crosslinking also implies that, to effectively combine impregnation with subsequent hot-pressing densification, the time interval between the two steps should be minimized to maintain modification efficiency.

To investigate the thermal curing behavior, the prepared solutions were heated at 120 °C for 12 h, and the resulting polymer morphologies are shown in Figure 3d. The 12PEGDGE sample primarily showed a reduction in volume due to water evaporation, while remaining in a liquid state. This further confirms that PEGDGE alone exhibits limited crosslinking ability, even under elevated temperatures. With the incorporation of AL, particularly at higher concentrations, the mixtures underwent visible curing and formed polymer structures. During heating, water evaporation resulted in polymer shrinkage, indicating network formation. These results confirm that PEGDGE can react with AL, forming a crosslinked network upon heating. The curing behavior of PEGDGE-AL shows its potential for in situ curing and fixation within the wood matrix through hot-pressing densification.

### 3.3. Physical Properties of the Wood Specimens

**Figure 4:**
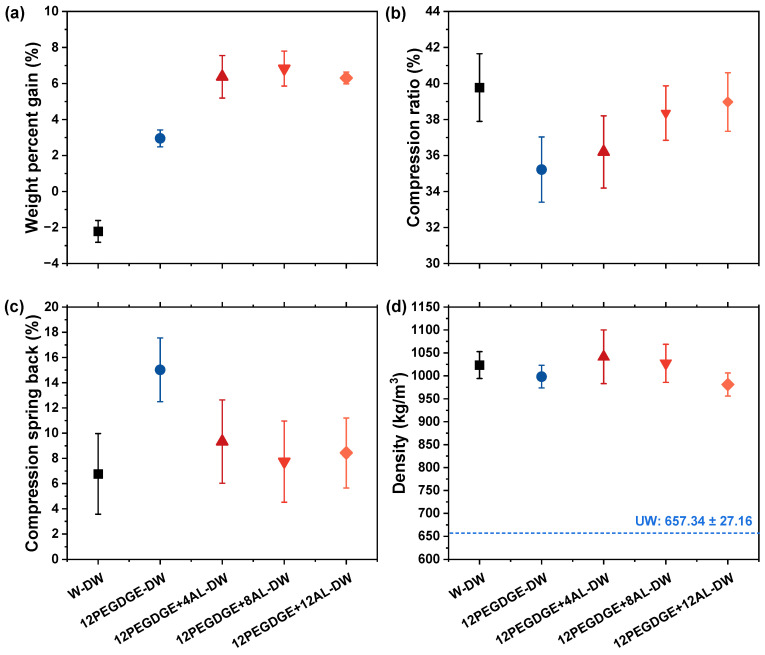
(**a**) Weight percent gain (WPG), (**b**) compression ratio (CR), (**c**) compression spring back (CSB), and (**d**) density of wood specimens impregnated with water and hot-pressed at 120 °C (W-DW) and wood specimens impregnated with PEGDGE-AL modifiers with different AL concentrations and hot-pressed at 120 °C (PEGDGE+AL-DW), where the short-dashed line represents the density of untreated wood (UW).

As shown in Figure 4a, the WPG of water-impregnated and densified wood (W-DW) was −2%, which can be attributed to the leaching of water-soluble extractives, such as starch [34], from rubberwood during water impregnation. Given the relatively low hot-pressing temperature employed in this study (120 °C), the thermal degradation of wood components is unlikely to have contributed greatly to this mass loss. For PEGDGE impregnated and densified wood, the WPG was relatively low (approximately 2%). This suggests that, in addition to the mass loss of modifiers during hot-pressing, the leaching of wood water-soluble extractives also contributed to the lower observed WPG, potentially affecting results. In contrast, the WPG of PEGDGE-AL impregnated and densified wood ranged from 6.3% to 6.8%, which similarly reflects the combined mass loss of both the wood extractions and the modifier during impregnation and hot-pressing. Given the high content of water-soluble extractives in rubberwood, the WPG may be underestimated. Nevertheless, it is evident that a portion of the modifier remains within the wood structure after densification.

As shown in Figure 4b, W-DW exhibited the highest compression ratio, approaching 40%. For PEGDGE-AL-impregnated and densified specimens, the CR gradually increased with higher AL content, ranging from 35% to 39%. The average CSB of W-DW was 6.8% (Figure 4c), indicating a partial shape recovery of thickness during curing process, caused by moisture evaporation and the release of compressive stress [35]. PEGDGE-impregnated and densified wood specimens showed higher CSB values than W-DW (15.0%), suggesting that PEGDGE alone was unable to form a cross-linked network within the wood structure, thus failing to fix the compressed deformation effectively. The addition of AL reduced the CSB values of PEGDGE-AL impregnated and densified wood, which ranged between 7.5% and 8.4%, indicating that the modifier had a limited fixation effect on the cell walls. This suggests that the compressive deformation of the wood is primarily attributed to the deformation of the cell walls themselves, and that the PEGDGE-AL modifiers mainly fixated in the cell lumens rather than being effectively incorporated into the cell wall matrix. Nevertheless, compared to PEGDGE treatment alone, the incorporation of AL reduced the CSB, indicating that AL contributed to the formation of a mechanically interlocked structure between the PEGDGE-AL network and the wood matrix during hot-pressing at 120 °C. This synergistic interaction not only reduced the compression spring back but also influenced the final compression ratio of the specimens, demonstrating the advantage of AL addition.

As shown in Figure 4d, hot-pressing densification reduced the volume of wood, which manifested macroscopically as an increase in density. However, no apparent difference in density was observed among the various impregnated and densified wood specimens. The density of W-DW was 1023 kg/m^3^, representing a 56% increase compared to unmodified wood (UW). Although PEGDGE-AL impregnation increased the overall mass of rubberwood, partial recovery during curing led to volumetric expansion, resulting in only slight variation in final density. The densities of PEGDGE-AL impregnated and densified wood ranged from 980 to 1040 kg/m^3^, showing a notable improvement compared to UW.

### 3.4. Surface Color of the Wood Specimens

**Figure 5:**
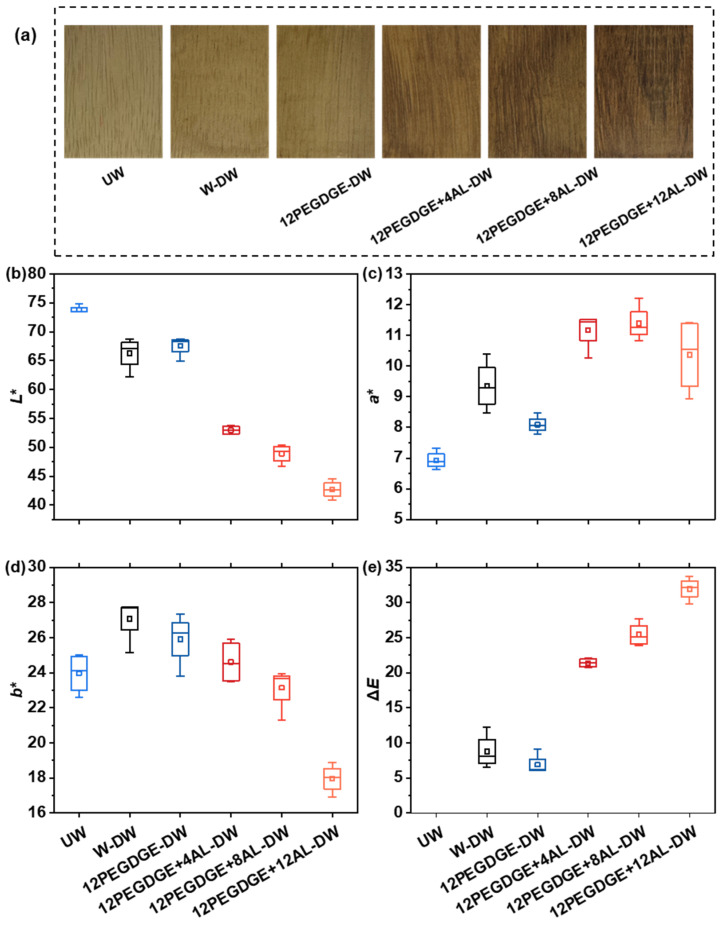
(**a**) Surface color of wood specimens and color parameters of wood specimens: (**b**) lightness, (**c**) red-green index, (**d**) yellow-blue index, and (**e**) color difference based on untreated wood. The bottom and top of the box plot exhibit the 25th percentile and 75th percentile, and the band near the middle of the box is the 50th percentile. The ends of the whiskers represent the 5th percentile and the 95th percentile.

The surface color and color difference parameters of the modified wood are presented in Figure 5. Compared to UW, the surface of W-DW became slightly darker. This darkening is attributed to the migration of water-soluble extractives toward the surface during hot-pressing, followed by their deposition [5,12,36]. The surface color of 12PEGDGE-DW remained close to that of UW, as PEGDGE is a colorless and transparent modifier and has minimal influence on the wood surface color. In contrast, the surface color of PEGDGE+AL-DW samples was visibly darker and more closely resembled that of lignin, with the color difference increasing proportionally to the AL content. This rapid color change can be ascribed to the abundance of chromophore groups in the aminated lignin, which likely migrated with moisture and deposited on the wood surface during hot-pressing [5].

From a commercial perspective, this change in surface color is advantageous, as the modified wood displays a rich dark-brown color, closely resembling that of high-end hardwoods. Therefore, PEGDGE-AL impregnation combined with low-temperature hot-pressing enhances the visual appeal of the wood, thereby adding aesthetic value and expanding its potential for decorative applications, especially for low-grade fast-growing wood species.

### 3.5. Morphological Analysis

**Figure 6:**
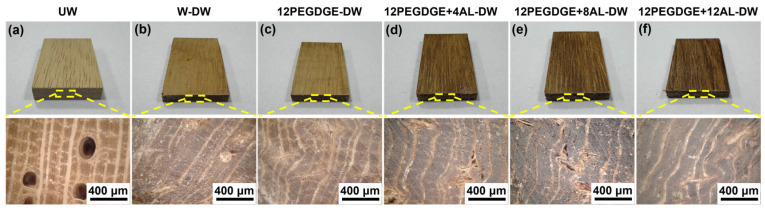
Digital images and ultra depth of field images of wood specimens: (**a**) UW, (**b**) W-DW, and (**c**–**f**) impregnated with PEGDGE-AL modifiers with different AL concentrations and hot-pressed at 120 °C.

The macroscopic and microscopic morphologies of untreated and densified rubber wood specimens were analyzed, as shown in Figure 6. Typical porous structures such as vessels, wood fibers, and wood rays are clearly visible in the untreated rubberwood. In water impregnated and densified wood (W-DW), the vessels and wood fiber cell lumens are compressed after hot-pressing densification, resulting in a dense wood structure. However, some uncompressed pores remain visible. The morphology of the 12PEGDGE impregnated and densified wood (12PEGDGE-DW) is similar to that of W-DW, with no apparent polymer distribution observed. This further confirms that PEGDGE alone does not undergo effective polymerization and fixation within the wood structure, which is consistent with the earlier analysis of its curing behavior.

In contrast, the PEGDGE-AL impregnated and densified wood (PEGDGE+AL-DW) exhibits changes in both color and compressed wood structure. After hot-pressing, some wood fiber lumens are visibly filled with the PEGDGE-AL polymer, which contributes to physical bonding between adjacent cell walls. However, certain pores remain visible within the compressed structure, which is in line with the partial recovery of deformation during the curing process, as indicated by compression spring back. Despite this, the impregnation of PEGDGE-AL solution effectively enhances the degree of densification. The fixation and partial filling of the polymer within the cell lumens are expected to contribute to the improved mechanical performance of the modified wood.

### 3.6. Mechanical Properties of the Wood Specimens

**Figure 7:**
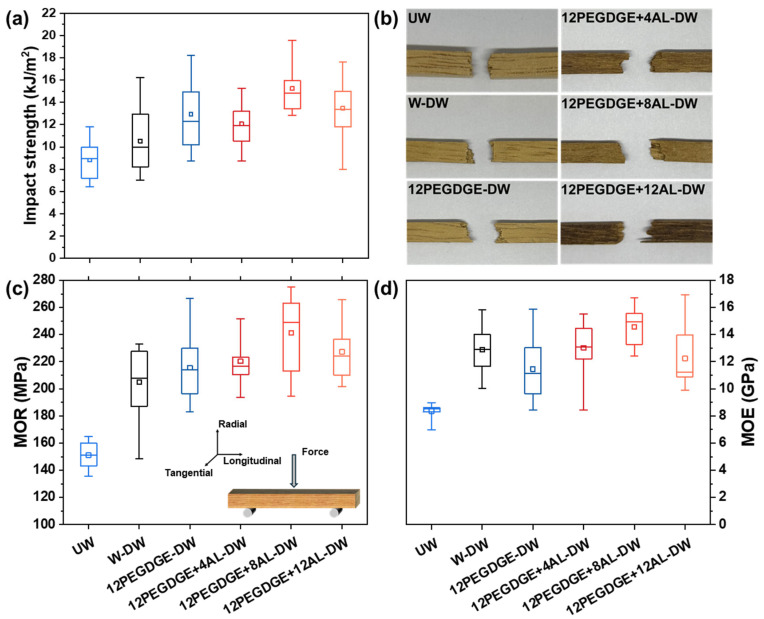
(**a**) Impact strength, (**b**) impact failure, (**c**) modulus of rupture (MOR), and (**d**) modus of elasticity (MOE) of untreated wood (UW), water-impregnated and densified wood (W-DW), and wood specimens impregnated with PEGDGE-AL modifiers with different AL contents and hot-pressed at 120 °C (PEGDGE+AL-DW). The bottom and top of the box plot exhibit the 25th percentile and 75th percentile, and the band near the middle of the box is the 50th percentile. The ends of the whiskers represent the 5th percentile and the 95th percentile.

The impact strength and flexural strength of untreated and densified wood specimens were evaluated, as shown in Figure 7. The impact strength of W-DW is slightly higher than that of UW. Further enhanced impact strength is observed in specimens impregnated with the PEGDGE-AL modifiers. Specifically, the impact strength of 12PEGDGE+8AL-DW and 12PEGDGE+12AL-DW reaches 15.2 and 13.5 kJ/m^2^, respectively, which are increased by approximately 73% and 52% compared to UW.

Failure analysis of the impact specimens (Figure 7b) reveals noticeable fiber pull-out in PEGDGE-AL impregnated and densified specimens, suggesting that the polymer is cross-linked and fixed within the wood structure via hot-pressing densification. The hot-pressing densification process effectively increases the number of wood cells per unit volume, resulting in a denser structure [37]. Further, the PEGDGE-AL polymer not only fills the lumens but also physically bonds adjacent cell walls, thereby increasing density and promoting a more uniform microstructure. This improved structural homogeneity facilitates enhanced impact strength.

The modulus of rupture (MOR) and modulus of elasticity (MOE) of the wood specimens were evaluated through three-point bending test. As shown in Figure 7c, the MOR of W-DW reached 205 MPa, approximately 36% higher than that of UW, which can be attributed to increased fiber content per unit volume resulting from densification, thereby enhancing stiffness and load-bearing capacity.

Further enhancements were observed in PEGDGE-AL impregnated and densified specimens. Among them, 12PEGDGE+8AL-DW exhibited the highest MOR of 241 MPa and MOE of 14.6 GPa, representing improvements of 59.5% and 74.6% over UW, and 18% and 13% over W-DW, respectively. All PEGDGE-AL modified specimens outperformed both UW and W-DW, owing to the formation of the cross-linked polymer network within the wood matrix under hot-pressing. This network effectively bonds cell walls and contributes to improved stress transfer and structural integrity.

These results confirm that PEGDGE-AL impregnation combined with hot-pressing at 120 °C effectively enhances the mechanical performance of rubberwood, particularly its impact and flexural strength, thereby expanding its potential for application in structural and load-bearing materials.

### 3.7. Dimensional Stability of the Densified Wood Specimens

**Figure 8:**
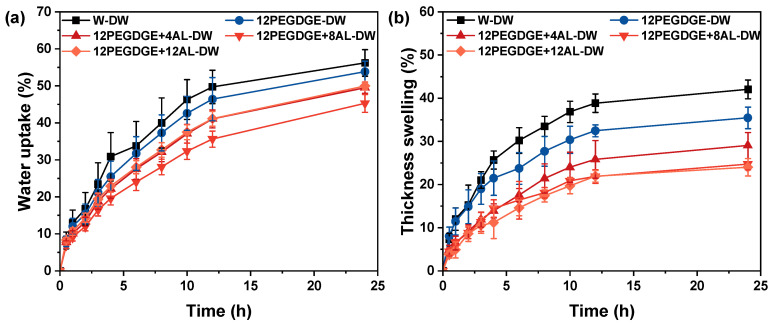
(**a**) Water uptake (WU) and (**b**) thickness swelling of the densified wood specimens.

The water uptake of densified wood specimens exhibited minor variations with a consistent overall trend (Figure 8a). The 24 h WU of W-DW reached 56.2%, which is due to thickness swelling while soaking in water (Figure 8b). This thickness swelling exposed additional internal pores, leading to increased water uptake. The incorporation of PEGDGE-AL slightly reduced the water absorption of densified wood. Among all, 12PEGDGE+8AL-DW exhibited the lowest WU (45.2%), representing a ~20% reduction compared to W-DW. However, differences in water uptake across specimens with varying AL content were relatively minor. This limited reduction can be explained by the chemical crosslinking of PEGDGE and AL. During the ring-opening curing process, each epoxy group reacts with a hydroxyl group, consuming one hydroxyl but simultaneously generating another new hydroxyl group [38]. As a result, the overall hydrophilicity of the system is not significantly reduced. As shown in Figure 8b, the thickness swelling (TS) of W-DW after 24 h of water immersion was as high as 42%, indicating poor water resistance and susceptibility to deformation recovery. In contrast, PEGDGE-AL-modified specimens exhibited lower TS values, which decreased progressively with increasing AL content. 12PEGDGE+8AL-DW showed the lowest TS (24.7%), demonstrating enhanced dimensional stability.

The improvements in both WU and TS are attributed to the in situ curing and fixation of PEGDGE-AL within the wood cell lumens during hot-pressing. The cross-linked polymer network bonds the adjacent cell walls, effectively blocking water transport pathways and reducing hydroxyl group accessibility of wood. Additionally, PEGDGE could also penetrate the cell walls and occupy the free volume of the wood cell wall, further limiting water uptake. Notably, when the AL content exceeds 8%, it further promotes the formation of an epoxy–aminated lignin cross-linking network. However, excessive AL can hinder impregnation efficiency, suggesting that 8% is an optimal addition level for achieving effective modification, making it more suitable for applications in humid environments.

## 4. Conclusions

In this study, an epoxy–aminated lignin (AL) modifier was successfully applied in the impregnation of fast-growing rubberwood, enabling effective enhancement of wood properties at a hot-pressing temperature as low as 120 °C. At this temperature and with an AL content of 8%, the wood specimens (denoted as 12PEGDGE+8AL-DW) demonstrated improvements in both mechanical performance and dimensional stability. Specifically, the impact strength of 12PEGDGE+8AL-DW reached 15.2 kJ/m^2^, corresponding to increases of 72% over unmodified wood (UW) and 44% over W-DW. Additionally, the MOR and MOE reached 241 MPa and 14.6 GPa, representing enhancements of 59.5% and 74.6% over UW, and 18% and 13% over W-DW, respectively. The 24 h water uptake and thickness swelling were 45.2% and 24.7%, representing reductions of 11% and 43% compared to water-impregnated and densified wood (W-DW). These findings indicate that the epoxy–aminated lignin cross-linked network effectively improves both mechanical properties and dimensional stability of fast-growing wood under a relatively low hot-pressing temperature of 120 °C. This work offers a promising strategy for the value-added utilization of lignin and fast-growing wood, providing insight into low-temperature, energy-efficient wood modification techniques for future sustainable applications. The property improvements in this study are primarily attributed to the filling effect of the PEGDGE-AL modifiers. Future works could focus on establishing chemical cross-linking between the modifiers and the wood cell walls, which holds great potential for further enhancing the dimensional stability and biological durability of modified wood.

## Figures and Tables

**Table 1:** Composition and solid content of PEGDGE-AL solutions with varying AL concentrations.

Impregnation Solution	PEGDGE Concentration (%)	AL Concentration (%)	Solid Content of the Solution (%)
12PEGDGE	12	0	12
12PEGDGE-4AL		4	16
12PEGDGE-8AL		8	20
12PEGDGE-12AL		12	24

## Data Availability

The original contributions presented in this study are included in the article. Further inquiries can be directed to the corresponding author.
